# Does closed-loop automated oxygen control reduce the duration of mechanical ventilation? A randomised controlled trial in ventilated preterm infants

**DOI:** 10.1186/s13063-022-06222-y

**Published:** 2022-04-08

**Authors:** Ourania Kaltsogianni, Theodore Dassios, Anne Greenough

**Affiliations:** 1 Neonatal Intensive Care Centre, King’s College Hospital NHS Foundation Trust, 4th Floor Golden Jubilee Wing, Denmark Hill, London, SE5 9RS UK; 2 Department of Women and Children’s Health, School of Life Course Sciences, Faculty of Life Sciences and Medicine, King’s College London, London, UK; 3 Asthma UK Centre for Allergic Mechanisms in Asthma, King’s College London, London, UK; 4 NIHR Biomedical Centre at Guy’s and St Thomas NHS Foundation Trust and King’s College London, London, UK

**Keywords:** Closed loop automated oxygen control, Preterm infants, Mechanical ventilation, Intermittent hypoxemia and hyperoxemia

## Abstract

**Background:**

Many preterm infants require supplemental oxygen in the newborn period but experience frequent fluctuations of their oxygen saturation levels. Intermittent episodes of hypoxia or hyperoxia increase the risk of complications. Compliance with achievement of oxygen saturation targets is variable, and the need for frequent adjustments of the inspired oxygen concentration increases workload.

Closed-loop automated oxygen control systems (CLAC) improve achievement of oxygen saturation targets and reduce both episodes of hypoxia and hyperoxia and the number of manual adjustments. This study investigates whether CLAC compared with manual oxygen control reduces the duration of mechanical ventilation in preterm infants born at less than 31 weeks of gestation.

**Methods:**

This randomised controlled trial performed at a single tertiary neonatal unit is recruiting 70 infants born at less than 31 weeks of gestational age and within 48 h of initiation of mechanical ventilation. Infants are randomised to CLAC or manual oxygen control from recruitment until successful extubation. The primary outcome is the duration of mechanical ventilation, and secondary outcomes are the percentage of time spent within target oxygen saturation ranges, the time spent in hypoxia or hyperoxia, the number of manual adjustments required, the number of days on oxygen, the incidence of bronchopulmonary dysplasia and the length and cost of neonatal unit stay. The study is performed following informed parental consent and was approved by the Yorkshire and the Humber-Sheffield Research Ethics Committee (protocol version 1.1, 13 July 2021).

**Discussion:**

This trial will investigate the effect of CLAC on the duration of mechanical ventilation, which is an important clinical outcome as prolonged mechanical ventilation is associated with important adverse outcomes, such as bronchopulmonary dysplasia.

**Trial registration:**

ClinicalTrials.Gov NCT05030337. Registered on 17 August 2021

**Supplementary Information:**

The online version contains supplementary material available at 10.1186/s13063-022-06222-y.

## Key study contacts

**Table:** 

Chief investigator	Professor Anne Greenough Neonatal Intensive Care Unit King’s College Hospital, SE5 9RS 02071887188 anne.greenough@kcl.ac.uk
Study coordinator	Professor Anne Greenough Neonatal Intensive Care Unit King’s College Hospital, SE5 9RS 02071887188 anne.greenough@kcl.ac.uk
Sponsor	Professor Reza Razavi Director of Research Management & Director of Administration (Health Schools), Room 5.31, James Clerk Maxwell Building, 57 Waterloo Road, London SE1 8WA Tel: + 44 (0)2078483224 Email: reza.razavi@kcl.ac.uk PA email: susan.dickson@kcl.ac.uk
Joint-sponsor(s)/co-sponsor(s)	Rahman Ahmed Research and Innovation Governance Manager King’s College Hospital NHS Foundation Trust 161 Denmark Hill, London, SE5 8EF e-mail: rahman.ahmed1@nhs.net
Funder(s)	The equipment was provided by SLE.
Key protocol contributors	Dr Ourania Kaltsogianni, Neonatal Grid Trainee and research fellow in Neonatal Medicine, King’s College London Dr Theodore Dassios, Consultant Neonatologist, King’s College Hospital, London
Committees	n/a

## Trial registration dataset

**Table:** 

Primary registry and trial identifying number	ClinicalTrials.Gov, NCT05030337
Date of registration	17/08/2021
IRAS no	297749
Source of monetary or material support	King’s College Hospital (KCH)/King’s College London (KCL)
Follow-up duration (if applicable)	Participants will be followed up until their discharge from the neonatal unit
Contact for public/scientific queries	Professor Anne Greenough Neonatal Intensive Care Unit King’s College Hospital, SE5 9RS 02071887188 anne.greenough@kcl.ac.uk
Public title	Optimising ventilation in preterms with closed-loop oxygen control
Scientific title	Does closed-loop automated oxygen control reduce the duration of mechanical ventilation? A randomised controlled trial in ventilated preterm infants
Country of recruitment	United Kingdom
Health condition(s) or problem(s) studied	Prematurity, mechanical ventilation, hypoxia, hyperoxia
Interventions	Closed-loop automated oxygen control (intervention group)
	Manual oxygen control (control group)
Key inclusion/exclusion criteria:	Inclusion criteria: preterm infants < 31 weeks gestation within 48 h of initiation of mechanical ventilation
	Exclusion criteria: infants > 31 weeks gestation or with major congenital anomalies
Study type	Randomised controlled trial
Date of first enrolment	05/09/2021
Target sample size	70
Recruitment status	Recruiting
Primary outcome	Duration of mechanical ventilation
Key secondary outcomes	Percentage of time spent in target saturation range, time spent in hypoxia and hyperoxia, number of manual adjustments required, number of days on oxygen, diagnosis of BPD, length and cost of neonatal stay

## Role of study sponsor and funder

King’s College London will take primary responsibility for ensuring the study design meets appropriate standards and uses proper conduct and reporting. King’s College London also provides insurance cover to provide for payment of damages or compensation in respect of any claim made by a research subject for bodily injury arising out of participation in a clinical trial or healthy volunteer study with certain restrictions.

King’s College Hospital takes responsibility for arranging the initiation and management of the research and will ensure that appropriate standards, conduct and reporting are adhered to with regards to its facilities and staff involved with the study. King’s College Hospital will also undertake the governance review for the project and provide cover for clinical negligence by any of its staff undertaking the research.

## Roles and responsibilities of study management committees, groups, and individuals

There are no committees or steering groups involved in the management of this study, but all the neonatal consultants have oversight of the study. The chief investigator and sponsor will monitor and audit the conduct of this research and will ensure that appropriate standards and reporting are adhered to (p6, 59–60). All the neonatal consultants have oversight of the study (p6, 70–71). In addition to this, an independent researcher not involved in the project will review trial data upon recruiting half of planned patients. The researcher will evaluate differences in the primary outcome to decide whether enough data have been gained at that point. The research team will remain blinded to the results of the interim analysis unless there are any significant findings (p12, 204–208). This is a non-blinded, randomised, superiority trial.

## Protocol contributors

This study has been designed by Professor Anne Greenough and Dr Theodore Dassios. Dr Ourania Kaltsogianni, neonatal grid trainee and research fellow, will conduct the study and primarily perform data analysis and interpretation, manuscript writing and dissemination of results with support from both supervisors.

## Background

Seven to eight per cent of all infants are born prematurely [1] and many require respiratory support in the newborn period [2]. Although such support can be life-saving, preterm infants who require mechanical ventilation and oxygen therapy frequently develop complications [3]. The most common adverse outcome is bronchopulmonary dysplasia (BPD), and importantly prematurely born infants can suffer chronic respiratory morbidity including troublesome respiratory symptoms, lung function abnormalities and exercise intolerance even in adolescence and adulthood [4]. Other complications include intracerebral haemorrhage which can result in cerebral palsy [5] and retinopathy of prematurity (ROP) which can cause blindness [6].

Ventilated neonates frequently require supplemental oxygen, but its use must be carefully monitored due to associated complications. Hyperoxia leads to the development of reactive oxide species (ROS) and increases the risk of bronchopulmonary dysplasia and retinopathy of prematurity [7, 8]. On the other hand, hypoxia increases morbidity and mortality [9, 10]. Therefore, oxygen saturation levels (SpO_2_) are continuously monitored in clinical practice and used to guide adjustments to the inspired oxygen concentration (FiO_2_), which are traditionally made manually by neonatal practitioners. Maintaining oxygen saturations in the target range can be difficult as preterm neonates experience frequent fluctuations with as many as 600 intermittent hypoxic episodes in a week documented in one study [11]. Moreover, prolonged hypoxemic episodes have been associated with increased risk of death after 36 weeks’ postmenstrual age or disability at 18 months corrected age including motor impairment, cognitive or language delay, severe hearing loss or bilateral blindness [12]. Compliance with achievement of SpO_2_ targets is variable within the same patient over time, as well as between patients and centres [13]. Furthermore, target achievement decreases as the number of patients per nurse increases [14].

Closed-loop automated oxygen control (CLAC) systems monitor SpO_2_ values in real-time to calculate and make an adjustment to the FiO_2_ patient delivery without any human intervention [15]. These systems may provide a solution for the low compliance with achievement of target oxygen levels and reduce the need for manual adjustments and hence the workload, as well as reducing complications. Several algorithms exist to calculate the FiO_2_ adjustment in relationship to the SpO_2_ changes in infants with different severities of respiratory disease [16]. An example is the Oxygenie Auto-O_2_ software (SLE) that uses a proportional-integral-derivative control algorithm, and it has been shown to be effective in improving compliance with target achievement in preterm infants, reducing episodes of hypoxia or hyperoxia and the need for manual adjustments to supplementary oxygen [17]. In a randomised crossover study, using that device we demonstrated that preterm ventilated infants experienced fewer prolonged desaturations during the closed-loop automated oxygen control period and spent an increased percentage of time within their target SpO_2_ range with fewer manual adjustments to the inspired oxygen concentration [18]. A review of 16 studies highlighted that the studies of CLAC were very heterogenous for design, population size and device used [15]. Moreover, the majority of those studies had a crossover design and limited data available for analysis. Two reviews emphasised that none of the previous studies had demonstrated whether the clinical outcomes of preterm infants were improved [15, 19]. A subsequent literature review including 18 studies [19] highlighted that CLAC was consistently associated with an increased percentage of time spent within the target oxygen saturation range with fewer manual adjustments to the FiO_2_ and was effective in infants on non-invasive respiratory support or mechanically ventilated at a range of postnatal ages. The results appear to be consistent for all the control algorithms [19] and across different SpO_2_ target ranges [20, 21]. In addition, previous studies demonstrated that CLAC could facilitate earlier weaning of the inspired oxygen concentration when compared to manual control [22, 23], which is a major determinant of an infant’s readiness for extubation. It is, therefore, possible and our hypothesis that use of CLAC would reduce the duration of mechanical ventilation

The aim of this study is to explore if in prematurely born ventilated infants, the use of closed loop automated oxygen control compared to standard care reduced the duration of mechanical ventilation. That is an important clinical outcome as prolonged mechanical ventilation increases the risk of BPD and log-term comorbidities [24]. Our primary outcome is the duration of mechanical ventilation. Secondary outcomes are the percentage of time spent within target oxygen saturation ranges, the time spent in hypoxia or hyperoxia, the number of manual adjustments required by clinical staff, a diagnosis of BPD at 36 weeks post menstrual age and the length of intensive care stay.

## Methods

This is a non-blinded, randomised controlled trial. Infants are allocated to parallel groups in a 1:1 ratio.

### Setting

The setting is a single tertiary neonatal unit at the King’s College Hospital NHS Foundation Trust, London, UK. The unit has previous experience on the use of closed-loop automated oxygen control in the context of trials.

### Inclusion criteria

Inclusion criteria are as follows:

1. Infants delivered at less than 31 weeks of gestational age requiring mechanical ventilation
2. Recruitment within 48 h of initiation of mechanical ventilation

### Exclusion criteria

1. Infants delivered above 31 weeks of gestational age
2. Infants with major congenital abnormalities

### Recruitment

Parents or legal guardians of all eligible infants are initially approached by the clinical team taking care of the infant and if they agree, by a researcher, within 24 h of initiation of mechanical ventilation. The parents are provided with an information sheet about the study. The researchers answer questions and respond to any concerns in a face-to-face meeting and obtain written informed consent. The study was approved by the Health Research Authority and by the Yorkshire and the Humber-Sheffield NHS Research Ethics Committee.

### Randomisation

The randomisation sequence is generated by using an online randomisation generator and concealed in sealed opaque envelopes. This is done by a person independent of the research team who is not involved in the study.

### Enrolment

Infants will be enrolled in the study by the research team within 48 h of initiation of mechanical ventilation following parental consent (supplied as attachment). Infants are randomised to one of the study arms by opening the next envelope.

### Blinding

The study is not blinded. Decisions regarding ongoing care, for example ventilator settings and interventions such as blood gases and chest radiographs, are made by the clinical team as per the neonatal unit’s guidelines.

### Intervention

At enrolment, participants are randomised to receiving either closed-loop automated oxygen control (intervention group) or manual control of the inspired oxygen concentration. All infants are ventilated using the SLE6000 ventilators and ventilation settings are manually adjusted by the clinical team as per the unit’s protocol. In addition to standard care, infants in the intervention group are connected to the Oxygenie Auto-O_2_ software (SLE). This software uses oxygen saturation levels from the SpO_2_ probe attached to the neonate, which are fed into an algorithm, to automatically adjust the percentage of inspired oxygen to maintain oxygen saturations within the target range. Manual adjustments including the percentage of inspired oxygen are allowed at any point during the study, if deemed appropriate by the clinical team.

The nurse-to-patient ratio is according to the unit protocol that is determined on the patient’s acuity. Respiratory care will be provided for all infants by the clinical team as per the unit’s guidelines. All infants will be ventilated using the SLE6000 ventilators and ventilation settings will be adjusted manually following the unit’s protocol. The intervention will be performed in addition to standard care and manual adjustments will be allowed at any point during the study, if deemed appropriate by the clinical team (p10–11, 173–180).

**Fig. 1:**
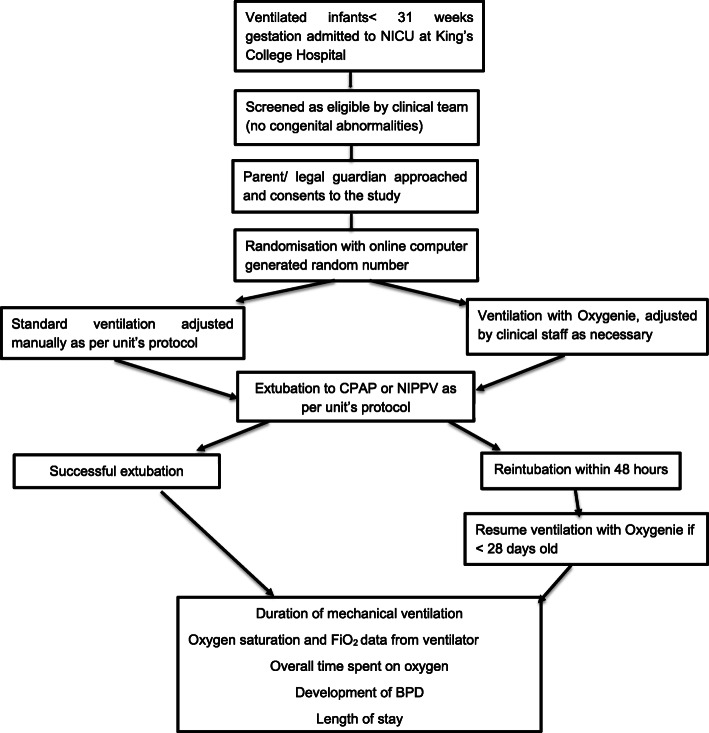
Trial flowchart (protocol v1.1, 13 July 2021)

Infants are studied from enrolment until successful extubation [25]. For infants who fail extubation and require reintubation within 48 h, they continue in their initial study arm if they are less than 28 days old. At KCH NICU, preterm infants with non-chronic respiratory pathology are extubated from a targeted tidal volume of 5 ml/kg, a peak inspiratory pressure at or below 18 cm H_2_O and a backup rate of 40 breaths per minute. FiO_2_ requirement should be less than 0.3. The infant should have good respiratory drive as well as blood gases. Therefore, for infants randomised to the intervention group, closed-loop oxygen delivery resumes. Preterm infants that remain ventilated beyond day 28 of life continue at their study arm (closed-loop automated oxygen control or manual oxygen control) till their first extubation attempt (see Fig. 1).

### Primary outcome measure

The primary outcome measure is the duration of mechanical ventilation. The primary outcome measure is the duration of mechanical ventilation from enrolment to the study until successful extubation. Extubation failure is defined as the need for reintubation within 48 h. For infants who fail extubation, they continue in their initial study arm if they are less than 28 days old, and the time they remain ventilated is counted towards the total duration of mechanical ventilation. For infants that remain ventilated beyond day 28 of life, the study continues till their first extubation attempt, and that period represents the primary outcome measure. Currently at KCH, the CLAC algorithm is used only for mechanically ventilated infants and in the context of clinical research studies. Hence, automatic oxygen control will be discontinued following extubation. Non-invasive ventilation modes will be delivered via the SLE6000 ventilators, and the control of the inspired oxygen concentration will be performed manually.

### Secondary outcomes

The secondary outcomes are the percentage of time spent within target oxygen saturation range, the time spent in hypoxia or hyperoxia, the number of manual adjustments required, the number of days on oxygen, the incidence of bronchopulmonary dysplasia at 36 weeks post menstrual age and the length and cost of neonatal unit stay. In line with the latest published evidence [10], our pulse oximetry oxygen saturation targets in preterm infants born at less than 31 weeks gestational age are 91 to 95% with alarm limits in the range of 90 to 98%. A researcher who is blinded to which arm the infant was randomised will undertake the analysis of the data. Retinopathy of prematurity will not be recorded as a secondary outcome, as our study is not powered to detect any significant differences in ROP outcomes between the two participating groups, but this outcome will be reported.

**Fig. 2:**
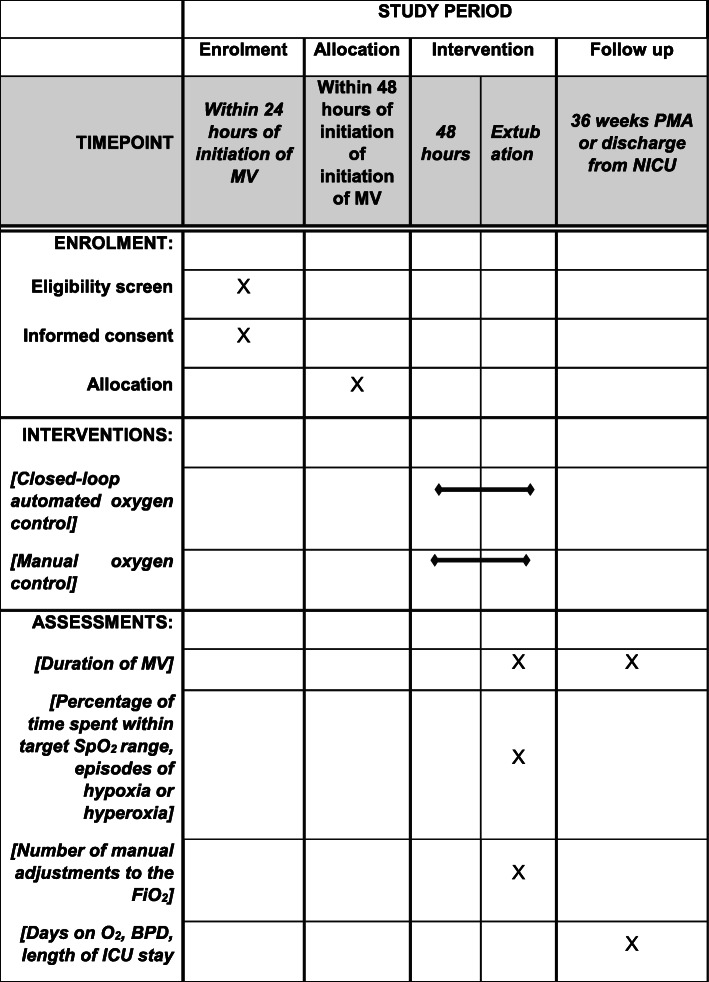
SPIRIT figure of trial interventions and timings

The order of study events is detailed in the Standard Protocol Items: Recommendations for Interventional Trials (SPIRIT) figure in Fig. 2. A SPIRIT checklist is also provided as an additional file and the protocol is based on the SPIRIT reporting guidelines [26].

### Sample size

In a cohort of 368 preterm infants born between 24 to 30 weeks of gestation, the standard deviation for the duration of mechanical ventilation was 3.83 days [27]. The median duration of ventilation in our NICU was 12 days (mean 19 days). Randomisation of 70 infants allows us to detect a decrease in the duration of mechanical ventilation of three days with a 90% power at the 5% significance level.

At the request of the ethics committee, an interim analysis will be carried out after half the sample has been recruited to review if enough data have been gained at that point. The analysis will be performed by an independent researcher not involved in the project to avoid introducing bias to our data. The research team will be blinded to the results of the interim analysis unless there are any significant findings.

### Data collection

Demographic and outcome data will be collected from the clinical records and recorded under a unique study identifier number. Basic epidemiologic parameters such as gestational age at birth, birth weight, corrected gestational age, postnatal age and weight at study enrolment, use of antenatal steroids, mode of delivery, doses of surfactant administered, birth plurality and associated comorbidities that may impact on the duration of mechanical ventilation, for example patent ductus arteriosus and infection, will be recorded for each infant. Oxygen saturation and FiO_2_ data will be downloaded from the ventilators. Paper data will be stored in a locked filling cabinet. Patient de-identified electronic data will be stored in password protected computers and on encrypted devices.

### Statistical analysis

Data will be compared between infants in the intervention group (closed-loop automated oxygen control) and infants in the control group (manual oxygen control). The data will be assessed for normality; Student’s *t* test will be used for normally distributed data and Mann-Whitney *U* test for skewed data. Categorical data will be assessed using the Fisher two-tailed exact test. Analysis will be undertaken using SPSS software (SPSS Inc., Chicago, IL, USA). Data analysis will be conducted on an intention-to-treat basis, and missing data will not be imputed. Analysis will be undertaken to a researcher blind to which arm the infants was randomised.

### Safety

All participants will receive standard care and monitoring by clinical staff. Ventilator settings will be adjusted and additional investigations such as blood gases and chest radiographs will be performed at the discretion of the clinicians. Therefore, there should not be any risk imposed to the intervention group with the addition of the closed-loop automated oxygen delivery system. The system does not mask large increased oxygen requirements between manual observation recordings, as it activates an alarm when there is an increase in the oxygen requirement ≥ 30% from the basal level alerting the clinical team. All serious adverse events will be recorded on a serious adverse event (SAE) form and will be emailed to the sponsor within one working day of the chief investigator (CI) becoming aware of the event. When the event is unexpected and thought to be related to the use of the device, this will be reported by the CI/sponsor to the ethics and Health Research Authority within 15 days.

### Protocol compliance

Any accidental protocol deviations will be appropriately documented and reported to the chief investigator and sponsor immediately. The chief investigator and sponsor will monitor and audit the conduct of this research. For any amendment to the study, the chief investigator or designee, in agreement with the sponsor, will submit information to the appropriate body in order for them to issue approval for the amendment (p13, 246–249).

### Research Ethics Committee (REC) and other regulatory review and reports

Before the start of the study, a favourable opinion was sought from a REC for the study protocol and other relevant documents (informed consent forms and patient information leaflets).

### Regulatory review and compliance

For any amendment to the study, the chief investigator or designee, in agreement with the sponsor, will submit information to the appropriate body in order for them to issue approval for the amendment.

### Data protection and patient confidentiality

The General Data Protection Regulation and Data Protection Act 2018 will be adhered to. Data will be de-identified before being entered in a secure database. A unique study identifier number will be issued to each participant on enrolment into the study. That number will be used in all subsequent data collection forms. Patient paper data will be stored in a locked filing cabinet in a room that is only accessible to the research team and that is based at the Neonatal Intensive Care Unit facilities at King’s College Hospital. Patient de-identified electronic data will be stored on encrypted university computers or memory stick devices, both of which require user identification and password verification.

### Archiving

At the end of the trial, all essential documentation will be archived securely by the CI for a minimum of 25 years from the declaration of the end of the trial. Essential documents include those which enable both the conduct of the trial and the quality of the data produced to be evaluated and show whether the site complied with all applicable regulatory requirements. All archived documents will continue to be available for inspection by appropriate authorities upon request.

### Indemnity

King’s College London indemnity applies for insurance/indemnity to meet the potential legal liability of the sponsor for harm to participants arising from the design and management of the research. NHS indemnity scheme applies for insurance/indemnity to meet the potential legal liability of the investigators arising from harm to participants in the conduct of the research.

### Access to the final study dataset

The individuals in the study will be notified of the outcome of the study as below. The investigators will have access to the final study dataset. Participants will be informed that anonymised data may be shared with other researchers for research purposes only.

### Dissemination policy

On completion of the study, the data will be analysed and a final study report will be prepared that could be accessed via the sponsor. The study will be presented at research meetings at the Neonatal Intensive Care Unit at King’s College Hospital as well as university meetings at King’s College London. Anonymised study data will be presented at conferences and published by the investigators in peer reviewed journals. Participants will be notified of the outcome of the study via provision of the publication and an accompanying newsletter.

### Authorship eligibility guidelines and any intended use of professional writers

The final study authors will include Dr Ourania Kaltsogianni, Dr Theodore Dassios and Professor Anne Greenough. Professional writers will not be used.

### Intellectual property

All intellectual property rights and know-how in the protocol and in the results arising directly from the study shall belong to KCL.

## Discussion

This study will compare the effectiveness of closed-loop automated oxygen control to manual oxygen control in preterm ventilated infants born at less than 31 weeks completed gestation. Previous studies in preterm infants demonstrated that CLAC improved the percentage of time spent within an assigned oxygen saturation target range [18, 20–23], but it remains unknown whether that improvement has any positive long-lasting effects on clinical outcomes. A retrospective cohort study comparing outcomes pre and post implementation of CLAC as standard care for preterm infants did not demonstrate any significant effect on morbidity or mortality at hospital discharge [28] but was associated with a significant reduction in the duration of mechanical ventilation. The study, however, was limited by its retrospective design and with several changes to neonatal care during the study span. Furthermore, it was not sufficiently powered to detect outcomes including BPD, ROP, necrotising enterocolitis (NEC), intraventricular haemorrhage (IVH) and periventricular leukomalacia (PVL), and the time infants received CLAC was not recorded, and the authors concluded that their results may have been biased by several factors not accounted for. This randomised controlled trial will provide more robust evidence on the effect of CLAC on the duration of mechanical ventilation that is related to long-term complications [24] and therefore may demonstrate if CLAC could improve long term respiratory outcomes for preterm infants. That will be essential knowledge before the intervention is implemented into standard care.

Participants are enrolled in our study within 48 h of initiation of mechanical ventilation. This enables a reasonable time frame to approach parents and obtain informed consent on participation. Infants are randomised to CLAC or manual oxygen control from recruitment to the study until they are successfully extubated. In comparison with the previous studies that mostly had a randomised crossover design and a short duration [17, 20, 29, 30], our study will provide more data points available for analysis and will explore the effect of CLAC during the different stages of evolving lung disease in preterm infants.

Over the past decade, increased knowledge of the risks related to mechanical ventilation and advances in non-invasive respiratory support have resulted in the reduction in the duration of mechanical ventilation [31]. Nevertheless, historical data from our unit for the period 2015–2020 demonstrated that the median duration of mechanical ventilation for preterm infants born less than 31 weeks gestation was 12 days. Hence, our study is powered to detect a decrease in the duration of mechanical ventilation of 3 days between the two treatment groups.

In conclusion, if this study demonstrates that CLAC is associated with earlier extubation and a shorter duration of mechanical ventilation, that could have significant impact on long-term respiratory outcomes of preterm infants.

## Trial status

At the time of submission, this trial has been approved by the NHS Research Ethics Committee and the Health Research Authority and is recruiting participants.

## Supplementary Information

**Additional file 1.**

**Additional file 2.**

**Additional file 3:** SPIRIT checklist for trials

## Data Availability

OK, TD and AG will have access to the final trial dataset. There are no contractual agreements that limit such access for all three investigators.

## Abbreviations

BPD: Bronchopulmonary dysplasia
CI: Chief investigator
CLAC: Closed-loop automated oxygen control
FiO2: Fraction of inspired oxygen concentration
IVH: Intraventricular haemorrhage
KCH: King’s College Hospital
KCL: King’s College London
NEC: Necrotising enterocolitis
PVL: Periventricular leukomalacia
ROP: Retinopathy of prematurity
ROS: Reactive oxide species
SpO_2_: Oxygen saturation levels
