# Supplementary material for: Does closed-loop automated oxygen control reduce the duration of mechanical ventilation? A randomised controlled trial in ventilated preterm infants
Source: Trials. 2022 Apr 8;23:276. doi: 10.1186/s13063-022-06222-y (PMC8994422; doi:10.1186/s13063-022-06222-y)
Supplement: Supplementary file 1 — Additional file 1. [file 13063_2022_6222_MOESM1_ESM.doc]

**Parents’ Information Sheet**

**Title of Study:** **Optimising ventilation in preterms with closed-loop oxygen control**

**Name of Researchers:** Professor Anne Greenough, Professor of Neonatology and Clinical Respiratory Physiology

Dr Theodore Dassios, Consultant Neonatologist

Dr Ourania Kaltsogianni, Research Fellow in Neonatal Medicine

We understand that this is a difficult time for you as your baby is in Intensive Care, but we would like you to consider taking part in our research study which will contribute to an MD Research degree. Before you decide we would like you to understand why the research is being done and what it would involve for your baby. One of our team will go through the information sheet with you and answer any questions you have. Please ask as many questions as you like and feel free to discuss the research with other people.

Part 1 of this document tells you the purpose of this study and what will happen to your child if you agree to take part. Part 2 gives you more detailed information about the conduct of the study. Ask us if there is anything that is not clear.

**Part 1:**

**What is the purpose of the study?**

Babies born earlier than their due date may need to be connected to a ventilator to help them breathe. In most cases, they also need extra oxygen to keep their brain and organs healthy. Mechanical ventilation is life-saving for those babies, but it needs to be kept as brief as possible because prolonged ventilation damages the lungs. Giving to the baby too much or too little oxygen may cause problems with eyesight and lung disease. For that reason, we monitor the baby’s oxygen levels carefully and adjust the amount of oxygen we give them as often as required.

Oxygen levels are monitored via a small probe placed on the baby’s hands or feet. The probe provides a signal to a monitor where we can read the oxygen levels and adjust manually the amount of oxygen provided to the baby. That amount changes frequently and it may be difficult to keep levels correct all the time. For that reason, new computer software has been developed called ‘closed loop automated oxygen control’ that automatically adjusts the amount of oxygen a baby gets according to their current levels. This software has already been studied in our unit and we found that it helps keep babies in the right amount of oxygen for more of the time. Also, the amount of oxygen provided is appropriately reduced more quickly and that could possibly help babies come off the ventilator sooner.

With this study we want to compare the time babies spend on the ventilator when we use the software to automatically monitor their oxygen levels with those babies whose oxygen is adjusted manually by the nurses and doctors. That could help us understand if the use of the software helps babies come off the ventilator sooner and the risk of damage to their lungs and other organs is reduced.

**Why has my baby been chosen?**

We will be approaching parents of all babies born earlier than 31 weeks gestation that are on mechanical ventilation to take part in the study.

**Does my baby have to take part?**

No. If you do not want to take part, this will in no way affect any care your baby may receive. If you agree for your baby to be included but later change your mind, you can withdraw from this study at any time without explanation.

**What will happen to my baby if they take part?**

We will use a computer tool to decide whether your baby will have their oxygen levels monitored and adjusted manually or automatically. That process is called ‘randomisation’ and ensures that the decision is based on chance alone. For those babies that will have the software added to their ventilator, the amount of oxygen received will be adjusted automatically. Their oxygen levels though will still be monitored by a nurse and the nurse can still change the amount of oxygen they receive if required.

We do not need to take any extra blood tests or do x-rays at all. Babies will only have an extra oxygen probe attached to their hands or feet. We will record the time all babies spend on the ventilator and any other problems they may develop during their stay.

**What are the possible risks of taking part?**

As we will be monitoring the oxygen levels of all babies and we will be able to adjust the amount of oxygen provided if needed, we don’t anticipate any danger arising from our study. Sometimes, the oxygen probe attached to the baby’s hands or feet may fall off and stop giving signal to the monitor. In this case, the monitor immediately generates an alarm to alert the baby’s nurse to re-site the probe. If there are any problems with the software, a member of the research team will be on site to help troubleshoot. If there are concerns about the safety of using the software, the baby’s nurse will be able to switch it off immediately and have sole control of the amount of oxygen the baby receives. The software is installed on the same ventilators we use for all babies and can be turned off without changing the ventilator or any of the tubing that connects the baby to the ventilator.

**What are the side effects of any treatment received whilst taking part?**

As we do not give any additional medicines than your baby would already be receiving, there would be no side effects.

**What are the possible benefits of taking part?**

Previous studies showed that the use of the software kept babies in the right amount of oxygen for more of the time and helped reduce the amount of oxygen provided more quickly. We may now understand if those babies do better in the long term and come off the ventilator sooner. Then we could use that software in our daily practice and help ventilated babies benefit in the future.

**What if there is a problem?**

Any concerns you may have about the study, or about the way in which you have been dealt with, will be addressed. More information about this is given in Part 2.

**Will my taking part in this study be kept confidential?**

Only the people involved in the study will have the original data. The findings will be published but no babies will be identified individually. However other individuals from the Trust may need to access the data for audit and monitoring.

**Parents’ Information Sheet**

**Title of Study: Optimising ventilation in preterms with closed-loop oxygen control**

**Part 2:**

**What if new information becomes available?**

If for any reason there is a recommendation that this practice should be changed, then that would happen for all babies in and out of the study.

**What happens if I don’t want to carry on with the study?**

You can choose to withdraw your baby from the study at any stage. This will not affect the care your baby receives in any way.

**What happens if something goes wrong?**

If you have a concern about any aspect of this study, you should ask to speak to the researchers who will do their best to answer your questions [Professor A. Greenough is the Principal Investigator, telephone number 020 7188 7188, e-mail: anne.greenough@kcl.ac.uk]. If you remain unhappy and wish to complain formally, you can do this through the King’s College Hospital Patients Advice and Liaison Service (PALS) on 020 3299 9000 extension 33601, Email: [kch-tr.PALS@nhs.net](mailto:kch-tr.PALS@nhs.net).

In the event that something does go wrong and you are harmed during the research you may have grounds for legal action for compensation against King’s College Hospital NHS Foundation Trust and/or King’s College London but you may have to pay your legal costs. The normal National Health Service complaints mechanisms will still be available to you (if appropriate).

**Will my taking part in this study be kept confidential?**

If you consent to take part in the research, any of the information collected about your baby may be inspected by the sponsor (including representatives of the sponsor). These inspections are solely for the purposes of the research and analysing the results. Your baby’s records may also be looked at by the regulatory authorities or ethics committees to check that the study is being carried out correctly.

The organisations listed above will keep information about your baby confidential and secure. Your baby’s name will not be used in any reports about the study and all data is stored in accordance with the principle of the Data Protection Act 2018. However, your baby’s hospital doctor may tell your GP about your baby’s participation if you agree for him/her to enter the study.

Personal data will be kept separately until your child is 25 years old that is standard practice for research involving children.

**Will my GP be informed?**

Your GP will be informed about the study via a letter and in your baby’s discharge summary.

**What will happen to the results of the study?**

We hope to present the results in scientific meetings in the UK and abroad and to publish the data in scientific journals. No personal information will be published at any stage.

**How we will use your data**

We will need to use information from your baby’s medical records for this research project.

This information will include your baby’s medical history such as gestation, birth weight, health problems and any investigations performed. We will also look at any problems you had or treatments received during pregnancy and around the time of delivery. People will use this information to do the research or to check the records to make sure that the research is being done properly.

People who do not need to know who your baby is will not be able to see his/her name or contact details. The data will have a code number instead.

We will keep all information about your baby safe and secure.

Once we have finished the study, we will keep some of the data so we can check the results. We will write our reports in a way that no-one can work out that your baby took part in the study.

**What are your choices about how your information is used?**
You can choose to withdraw you baby from the study at any time, without giving a reason, but we will keep information about your baby that we already have.

We need to manage your baby’s records in specific ways for the research to be reliable. This means that we won’t be able to let you see or change the data we hold about your baby.

**Where can you find out more about how your information is used?**

If you consent to take part we will use your data to deliver this project as described above in this Patient Information Sheet. If you would like to find out more please read the supplementary leaflet provided, entitled ‘**How we use your data’**’

This information is also available on the Health Research Authority website: [www.hra.nhs.uk/information-about-patients/](https://www.hra.nhs.uk/information-about-patients/).

**Who is organising the research?**

Professor Anne Greenough and Dr Theodore Dassios are leading this study. King’s College London is the sponsor of the study and King’s College Hospital acts as a co-sponsor.

**Who has reviewed this study?**

All research in the NHS is looked at by an independent group of people, called a Research Ethics Committee. This study has been reviewed and given favourable opinion by the XXX.

**Further information and contact details.**

Should you have any further questions regarding this study please ask one of the research team:

Professor Anne Greenough, e-mail: [anne.greenough@kcl.ac.uk](mailto:anne.greenough@kcl.ac.uk), tel: 020 7188 7188

Dr Theodore Dassios, e-mail: [theodore.dassios@kcl.ac.uk](mailto:theodore.dassios@kcl.ac.uk), tel: 020 3299 4644

Dr Ourania Kaltsogianni, e-mail: [ourania.kaltsogianni@nhs.net](mailto:ourania.kaltsogianni@nhs.net), tel: 020 3299 8492.

**Thank you very much for considering taking part in this study.**
