# Supplementary material for: Does closed-loop automated oxygen control reduce the duration of mechanical ventilation? A randomised controlled trial in ventilated preterm infants
Source: Trials. 2022 Apr 8;23:276. doi: 10.1186/s13063-022-06222-y (PMC8994422; doi:10.1186/s13063-022-06222-y)
Supplement: Supplementary file 2 — Additional file 2. [file 13063_2022_6222_MOESM2_ESM.doc]

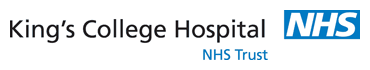

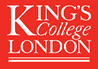


Neonatal Intensive Care Unit,

Main Reception: +44 (0)20 3299 3553

Study Number:

Participant Identification Number for this trial:

**CONSENT FORM**

**Title of Project:** **Optimising ventilation in preterms with closed-loop oxygen control**

Please initial box

1. I confirm that I have read the information sheet dated 13.07.21 (version 1.1) for the
   above study. I have had the opportunity to consider the information, ask questions and have
   had these answered satisfactorily.
2. I understand that my child’s participation is voluntary and that I am free to withdraw at any time
   without giving any reason, without my child’s medical care or legal rights being affected.
3. I understand that relevant sections of my child’s medical notes and data collected during
   the study, may be looked at by responsible individuals and King’s College Hospital Research

and Development Office or regulatory authorities, where it is relevant to my child taking part in this

research. I give permission for these individuals to have access to my child’s records.

1. I understand that the information collected about my child will be used to support
   other research in the future and may be shared anonymously with other researchers.
2. I give permission for my maternity notes and medical records to be accessed by the researchers

to collect essential information about the study as explained in the information sheet.

1. I understand that the data collected about my child will be kept securely for a minimum of 25 years.
2. I agree for my child’s GP to be informed about participating in the study.
3. I agree for my child to take part in the above study.

Name of child

Name of Participant Date Signature

Name of Person taking consent Date Signature
